# Supplementary material for: The prevalence of under nutrition and associated factors among pregnant women attending antenatal care service in public hospitals of western Ethiopia
Source: PLoS One. 2023 Jan 17;18(1):e0278180. doi: 10.1371/journal.pone.0278180 (PMC9844891; doi:10.1371/journal.pone.0278180)
Supplement: S1 Annex — (DOCX) [file pone.0278180.s001.docx]

## Annex I: English version Consent information sheet

Good morning/good afternoon. My name is ___________. We came from Wollega University College of Health science, Department of Public Health. We are working for an investigator doing his thesis for the partial fulfillment of master’s degree in public health. We would like to ask you few questions about factors affecting your nutritional status. This will help us to identify some of the barriers to good nutritional status outcomes based on your answer to our questions.

We will also take some measurements like mid upper arm circumference, height and weight from you. If you are interested, we can tell your measurements. You have full right to refuse, withdraw or completely reject part or all of your participation in the study. But we encourage your full participation as the answers you give on this form and your participation in taking your measurements are very important to this study.

We would like to assure you that all of your responses to our questions will be kept confidential throughout the study process. Any of your information you provide will be used only by the research team and by no means, be revealed to a third party. We will ask you questions and take measurements in a place where other people or conditions could not interfere.

We would like to assure you that your participation on this research will not affect any of your interest and other benefit that you get from any organization. We would be thankful if you spend some time with us answering questions related to the issues described above and cooperating in taking some measurements from you. The questions and measurements will take 30-40 minutes. May I get your permission to continue my interview?

1. Yes (continue), 2.No (stop).

**Data collectors**

Name_______________________ Sign_____ Date _________________

**Supervisor**

Name_______________________ Sign_____ Date _________________

## Annex II: English Version Questionnaires:

To assess magnitude of undernutrition and its associated factors among pregnant women attending ANC services at Public Hospitals of West Wollega Zone.

Name of Health facility _____________________________

Questionnaire serial Number (code)_____________ Date ________________

**Section 1: Questions about General Socio-demographic, socioeconomic, and environmental and cultural Characteristics**

Section 1.1 General Socio-demographic, socioeconomic and environmental characteristics of respondents

| S. No | Questions | Respond options | Remark |
| --- | --- | --- | --- |
| 1.1 | What is your Age | _______ years |  |
| 1.2 | Religion | 1 Muslim 2 Protestant  3 Orthodox 4 Catholic 5.Others (specify) _________ |  |
| 1.3 | Marital status | 1. Single 2. Married 3.Widowed  4. Divorced 5.Others (specify) _______________ |  |
| 1.4 | What is your residency? | 1 Urban  2 Rural |  |
| 1.5 | What is your educational status? | 1. Can’t read and write  2. Only Read & Write  3. Primary education (1-8)  4. Secondary education (9-10)  5. Preparatory (11-12)  6. Level and Diploma  7. Degree and above |  |
| 1.6 | What is your couples  educational status | 1. Can’t read and write  2. Only Read & Write  3. Primary education (1-8)  4. Secondary education (9-10)  5. Preparatory (11-12)  6. Level and Diploma  7. Degree and above |  |
| 1.7 | What is your Occupation? | 1. Gov’t Employee 2.Farmer 3.Merchant 4.Housewife 5.Daily laborer 6.Student 7.Non-gov’t employee 8.Specify others­­­­­­­­­­­­­­ |  |
| 1.8 | What is your couple’s occupation? | 1. Gov’t employee 2.Farmer 3.Merchant 4. Daily laborer 5.Student 6.Non-gov’t employee 7..Others (specify)­­­­­­­­­­­­­­_________ |  |
| 1.9 | What is the family size of  your Household? | ___________ |  |
| 1.10 | How many under five children's did you have in the household | ___________ |  |
| 1.11 | What is your monthly  Family income | ___________ETB |  |
| 1.12 | Do you have latrine facility  at your home | 0 = No  1 = Yes |  |
| 1.13 | Source of drinking water | 1. Piped water 2. Protected well 3. Unprotected well 4. Protected spring 5. Unprotected spring 6. Rainwater 7. Surface water 8. Bottled water |  |

Section 1.2 Questions to identify respondent’s sociocultural characteristics or decision- making autonomy

| S.NO | Question | Response | Remark |
| --- | --- | --- | --- |
| 1.14 | Would you decide by your own self to go health institution when you become sick? | 0 = No  1 = Yes |  |
| 1.15 | Would you decide by your own self for major house hold purchase | 0 = No  1 = Yes |  |
| 1.16 | Would you decide by your own self for major house hold expenditures | 0 = No  1 = Yes |  |
| 1.17 | Would you decide by your own self for visiting friends and participating meeting/celebrations | 0 = No  1 = Yes |  |

**Section 2: Reproductive, medical, health care, and behavioral characteristics of respondents**

| S.NO | Question | Response | Remark |
| --- | --- | --- | --- |
| 2.1 | What was your age at your first marriage? | ­­­­­___________________ |  |
| 2.2 | Number of pregnancy (Gravida) |  |  |
| 2.3 | Number of previous birth (parity) | __________________ | Skip if Q2.2 is 1) |
| 2.4 | Birth interval | 1. <2 years  2. 2 to 4 years  3. >= 4 years |  |
| 2.5 | Gestational Age | _____________ |  |
| 2.6 | Is your current pregnancy intended? | 0 = No  1 = Yes |  |
| 2.7 | Current ANC Visit number | 1. 1^st^ Visit  2. 2^nd^ Visit  3. 3^rd^ visit  4. 4^th^ visit |  |
| 2.8 | Have ever used any contraceptives before | 0 = No  1 = Yes |  |
| 2.9 | Do you get nutritional advice during pregnancy visit | 0 = No  1 = Yes |  |
| 2.10 | Do you take any IFA supplementation during current pregnancy | 0 = No  1 = Yes |  |
| 2.11 | Do you get Deworming supplementation during your current pregnancy | 0 = No  1 = Yes |  |
| 2.12 | History of any complication related to current or previous pregnancy | 0 = No  1 = Yes |  |
| 2.13 | Do you experience any illness recently/ within last 2 weeks | 0 = No  1 = Yes |  |
| 2.14 | Do you experience illness frequently | 0= No  1 = yes |  |
| 2.15 | Do you have any chronic illness lasting months or years? | 0= No  1= yes |  |
| 2.16 | Do you use any of the following substance (alcohol, Chat, Cigarettes, etc)? | 0 = No  1 = Yes |  |

3.2: Occurrence and Frequency of Household Food Insecurity of respondents

| S.NO | Question | Response options | Code |
| --- | --- | --- | --- |
| 1 | In the past four weeks, did you worry that your household would  not have enough food? | 0 = No (skip to Q2)  1=Yes |  |
| 1.a | How often did this happen? | 1 = Rarely (once or twice)  2 = Sometimes (3-10 times)  3 = Often (more than ten times) |  |
| 2 | In the past four weeks, were you or any HH member not able to  eat the kinds of foods you preferred because of a lack of resources? | 0 = No (skip to Q3)  1=Yes |  |
| 2.a | How often did this happen? | 1 =Rarely (once or twice)  2 =Sometimes (3-10 times)  3 = Often (more than ten times) |  |
| 3 | In the past four weeks, did you or any HH member have to  eat a limited variety of foods due to a lack of resources? | 0 = No (skip to Q4)  1 =Yes |  |
| 3a | How often did this happen? | 1 =Rarely (once or twice)  2 = Sometimes (3-10 times)  3 = Often (more than ten times |  |
| 4 | In the past four weeks, did you or any HH member have to  eat some foods that you really did not want to eat because of a  lack of resources to obtain other types of food? | 0 = No (skip to Q5)  1 = Yes |  |
| 4.a | How often did this happen? | 1 =Rarely (once or twice)  2 =Sometimes (3-10 times)  3 = Often (more than ten times) |  |
| 5 | In the past four weeks, did you or any HH member have to eat  a smaller meal than you felt you needed because there was not enough food? | 0 = No (skip to Q6)  1 =Yes |  |
| 5.a | How often did this happen? | 1 = Rarely (once or twice)  2 = Sometimes (3-10 times )  3 = Often (more than ten times) |  |
| 6 | In the past four weeks, did you or any other HH member have to  eat fewer meals in a day because there was not enough food? | 0 = No (skip to Q7)  1 =Yes |  |
| 6.a | How often did this happen? | 1 =Rarely (once or twice)  2 =Sometimes (3-10 times)  3 = Often (more than ten times) |  |
| 7 | In the past four weeks, was there ever no food to eat of any kind in your HH because of lack of resources to get food? | 0 = No (skip to Q8)  1 = Yes |  |
| 7.a | How often did this happen? | 1 = Rarely (once or twice)  2 =Sometimes (3-10 times)  3 =Often (more than ten times |  |
| 8. | In the past four weeks, did you or any HH member go to sleep  at night hungry because there was not enough food? | 0 = No (skip to Q9)  1 =Yes |  |
| 8.a | How often did this happen? | 1 = Rarely (once or twice)  2 =Sometimes (3-10 times)  3 =Often (more than ten times) |  |
| 9 | In the past four weeks, did you or any HH member go a whole  day and night without eating anything because there was not enough food? | 0 =No (skip to section 4.3)  1 = Yes |  |
| 9.a | How often did this happen? | 1 = Rarely (once or twice)  2 =Sometimes (3-10 times)  3 =Often (more than ten times) |  |

Section 3:3: Minimum Dietary Diversity Score of respondents

| Now, I will ask you the different food groups if you consumed in the last 24 hours? You will respond by saying Yes or No for each food group. (if there is any special diet ceremony in the house, please ask about the day before that day) | Yes (1)√ | No(0) √ | Code |
| --- | --- | --- | --- |
| 1. Grains, white roots and tubers, and plantains |  |  |  |
| 2. Pulses (beans, peas and lentils) |  |  |  |
| 3. Nuts and seeds |  |  |  |
| 4. Dairy |  |  |  |
| 5. Meat, poultry and fish |  |  |  |
| 6. Eggs |  |  |  |
| 7. Dark green leafy vegetables |  |  |  |
| 8. Other vitamin A-rich fruits and vegetables |  |  |  |
| 9. Other vegetables |  |  |  |
| 10. Other fruits |  |  |  |
| 11. Condiments and seasonings |  |  |  |
| 12. Other beverages and foods |  |  |  |

Section 3.4: Semi quantitative food frequency questionnaire (FFQ) to identify dietary intake of respondents

| Please, tell me in your average total use, during the past one month, of each specific food and drink | Once  per month | 2-3  per month | 1 per  Week | 2 per  week | 3-4  per week | 5-6  per week | daily | 2-3  per day | > 3 per  Day |
| --- | --- | --- | --- | --- | --- | --- | --- | --- | --- |
| **1. Cereals** (bread, pasta, macaroni, biscuits, cookies or any other foods made from millet, sorghum, maize, rice, wheat + insert local foods e.g. porridge or pastes or other locally available grains) |  |  |  |  |  |  |  |  |  |
| **2. Legumes, nuts, and seeds** (beans, peas, lentils, nuts, seeds or foods made from these) |  |  |  |  |  |  |  |  |  |
| **3. Dark green leafy vegetables** (sweet pepper, dark green/leafy vegetables, locally available vitamin-A rich leaves) |  |  |  |  |  |  |  |  |  |
| **4. Yellow-orange vegetable**  (pumpkin, carrots, or sweet potatoes that are yellow or orange inside +other locally available vitamin-A rich vegetables) |  |  |  |  |  |  |  |  |  |
| **5. White tubers and roots**  (Potatoes or foods made from roots.) |  |  |  |  |  |  |  |  |  |
| **6. Flesh meats** (beef, lamb, goat, chicken) |  |  |  |  |  |  |  |  |  |
| **7. Milk and milk products** (milk, cheese, yogurt or other milk products) |  |  |  |  |  |  |  |  |  |
| **8. Eggs** |  |  |  |  |  |  |  |  |  |
| **9. Fish** |  |  |  |  |  |  |  |  |  |
| **10. Oils and fats** (oil, fats or butter added to food or used for cooking) |  |  |  |  |  |  |  |  |  |
| **11. Sweets**(sugar, honey, sweetened soda or sugary foods such as chocolates, sweets or candies) |  |  |  |  |  |  |  |  |  |
| **12. Coffee/tea** (tea (black, green) or coffee) |  |  |  |  |  |  |  |  |  |

**Section 4: Anthropometric measurement of women attending ANC services at Public Hospitals of West Wollega Zone from February to March 2020.**

| S. No | Method | Measurement | Code |
| --- | --- | --- | --- |
| 1. | MUAC (cm) | _____cm |  |

I have finished my interview and if you have any question or suggestion you can raise, if not I ask you to put your signature confirming that this data represents you and your household.

I confirm that this data is mine: Date ___________________ Signature ____________

## Annex III: Enumerator’s Guide on category of different food groups

**Enumerators guide on data collection on dietary diversity**

Now I would like to ask you to describe everything that you ate or drank yesterday during the day or night, whether you ate it at home or anywhere else. Please include all foods and drinks, any snacks or small meals, as well as any main meals. Remember to include all foods you may have eaten while preparing meals or preparing food for others. Please also include food you ate even if it was eaten elsewhere, away from your home. Let us start with the first food or drink consumed yesterday.

Did you have anything to eat or drink when you woke? If yes, what? Anything else?*

Did you have anything to eat or drink later in the morning? If yes, what? Anything else?*

Did you eat or drink anything at mid-day? If yes, what? Anything else?*

Did you have anything to eat or drink during the afternoon? If yes, what? Anything else?*

Did you have anything to eat in the evening? If yes, what? Anything else?*

Did you have anything else to eat or drink in the evening before going to bed or during the night? If yes, what? Anything else?*

**Food lists with respective food group to insert in while the respondent says she consumed it**

| S.NO | Food group | Food items |
| --- | --- | --- |
| 1 | Grains, white roots and tubers | - Breads, Injera, porridge, and other foods made from barley, wheat, maize, millet, sorghum, teff, and - Potatoes, sweet potato, anchote, white elephant yam and other foods made from these. |
| 2 | Pulses | - Foods made from Beans ( broad bean, kidney beans, etc), peas (chick pea, cow pea, sweet pea, pigeon pea, etc), and lentils |
| 3 | Nuts and seeds | - Peanuts, Sesame seed, sunflower seed, etc |
| 4 | Milk and milk products | - Fresh whole milk, soft cheese, hard cheese, yoghurt |
| 5 | Meat, poultry, and fish | - Raw or processed meat got from beef, goat, sheep, chicken and fish |
| 6 | Eggs | - Chicken egg |
| 7 | Dark green leafy vegetables | - Cabbage, lettuce, kale |
| 8 | Other Vitamin A rich fruits and vegetables | - Carrots, pumpkin, red sweet pepper, ripe mango, ripe papaya |
| 9 | Other vegetables | - cauliflower, green pepper, tomato, key sir, onions |
| 10 | Other fruits | - Apple, Avocado, banana, lemon, orange, grapes |
| 11 | Condiments and seasonings | - Chili pepper, ginger roots, garlic, |
| 12 | Other beverages and foods | - Tea, coffee, alcohol, and sweetened drinks |
