# Supplementary material for: The prevalence of under nutrition and associated factors among pregnant women attending antenatal care service in public hospitals of western Ethiopia
Source: PLoS One. 2023 Jan 17;18(1):e0278180. doi: 10.1371/journal.pone.0278180 (PMC9844891; doi:10.1371/journal.pone.0278180)
Supplement: Attachment — Submitted filename: PONE_REV.pdf [file pone.0278180.s005.pdf]

PLOS ONE

The magnitude of under nutrition and associated factors among pregnant women attending Antenatal Care service in Public Hospitals of western Ethiopia.

--Manuscript Draft--

Manuscript Number:	PONE-D-21-23542
Article Type:	Research Article
Full Title:	The magnitude of under nutrition and associated factors among pregnant women attending Antenatal Care service in Public Hospitals of western Ethiopia.
Short Title:	The magnitude of under nutrition and associated factors among pregnant women in western Ethiopia.
Corresponding Author:	Lamessa Tafara, MPH<br>Oromia Regional Health Bureau<br>Enango, ETHIOPIA
Keywords:	
Abstract:	Background: Pregnancy constitutes states of considerable physiological stress, which cause increased nutritional demands. Under nutrition is a worldwide health issue, especially among pregnant women. Malnutrition during pregnancy can result in miscarriages, fetal deaths during pregnancy, pre-term delivery, and maternal mortality for both the mother and her fetus. Therefore, this research was aimed to assess the magnitude of under nutrition and associated factors among pregnant women attending antenatal care services at public hospitals of West Wollega Zone.<br>Objective: To assess the magnitude of under nutrition and associated factors among pregnant women attending Antenatal Care service in Public Hospitals of western Ethiopia.<br>Methods: Facility based cross-sectional study was conducted using systematic random sampling techniques. Interviewer administered structured questionnaire was used to collect the data and Mid-upper arm circumference, height and weight were measured to determine the magnitude of under nutrition. Then, the data were analyzed using logistic regression with 95% confidence interval (CI). Besides, the results of data analysis were presented using appropriate descriptive statistics.<br>Result: The magnitude of under nutrition among pregnant women was found to be 39.2% (95%CI: 35.7%, 42.6%). Rural residence [(AOR=1.97, 95% CI: (1.24, 3.14)], substance use [(AOR: 3.33, 95% CI: (1.63, 6.81)], low dietary diversity of women [(AOR= 7.56, 95% CI: (4.96, 11.51)], mildly food insecure household [(AOR= 4.36, 95% CI: (2.36, 8.79)] ,moderately food insecure household [(AOR= 3.71, 95%CI: (1.54, 8.79), and severely food insecure household [(AOR= 6.96, 95% CI: (3.15, 15.42)] were factors significantly associated with under nutrition.<br>Conclusion: The magnitude of under nutrition is very high among pregnant women. Rural residency, household food insecurity, dietary diversity and substance use were significant predictors of undernutrition among pregnant women. Therefore, all concerned bodies should made efforts to reduce the risk of under nutrition by reducing substance use and improving household food security there by to increase women's dietary diversity.
Order of Authors:	Lamessa Tafara, MPH
	Haile Bikila
	Ilili Feyisa
	Markos Desalegn
	Zalalem Kaba
Additional Information:	
Question	Response
Financial Disclosure	The author(s) received no specific funding for this work.

Powered by Editorial Manager® and ProduXion Manager® from Aries Systems Corporation

Enter a financial disclosure statement that describes the sources of funding for the work included in this submission. Review the submission guidelines for detailed requirements. View published research articles from *PLOS ONE* for specific examples.<br><br>This statement is required for submission and **will appear in the published article** if the submission is accepted. Please make sure it is accurate.<br><br>**Unfunded studies**<br>Enter: *The author(s) received no specific funding for this work.*<br><br>**Funded studies**<br>Enter a statement with the following details:<br>• Initials of the authors who received each award<br>• Grant numbers awarded to each author<br>• The full name of each funder<br>• URL of each funder website<br>• Did the sponsors or funders play any role in the study design, data collection and analysis, decision to publish, or preparation of the manuscript?<br>• **NO** - Include this sentence at the end of your statement: *The funders had no role in study design, data collection and analysis, decision to publish, or preparation of the manuscript.*<br>• **YES** - Specify the role(s) played.<br><br>* typeset	
**Competing Interests**<br><br>Use the instructions below to enter a competing interest statement for this submission. On behalf of all authors, disclose any competing interests that could be perceived to bias this work—acknowledging all financial support and any other relevant financial or non-financial competing interests.<br><br>This statement is required for submission and **will appear in the published article** if the submission is accepted. Please make	The authors have declared that no competing interests exist.

sure it is accurate and that any funding sources listed in your Funding Information later in the submission form are also declared in your Financial Disclosure statement.<br><br>View published research articles from *PLOS ONE* for specific examples.<br><br>**NO authors have competing interests**<br><br>Enter: *The authors have declared that no competing interests exist.*<br><br>**Authors with competing interests**<br><br>Enter competing interest details beginning with this statement:<br><br>*I have read the journal's policy and the authors of this manuscript have the following competing interests: [insert competing interests here]*<br><br>* typeset	
**Ethics Statement**<br><br>Enter an ethics statement for this submission. This statement is required if the study involved:<br><br>• Human participants<br>• Human specimens or tissue<br>• Vertebrate animals or cephalopods<br>• Vertebrate embryos or tissues<br>• Field research<br><br>Write "N/A" if the submission does not require an ethics statement.<br><br>General guidance is provided below. Consult the submission guidelines for detailed instructions. **Make sure that all information entered here is included in the Methods section of the manuscript.**	The study protocol was approved and permission was obtained from Wollega University. An official letter of co-operation was written to the selected Public Hospitals of West Wollega Zone Administration. Information on the studies, including purpose and procedures was given for participants. Written or verbal consent was obtained from each participant. In order to protect confidentiality, names or identifications were not included on the written questionnaires. Identification of the respondents was only through numerical codes.

Powered by Editorial Manager® and ProduXion Manager® from Aries Systems Corporation

**Format for specific study types**<br><br>**Human Subject Research (involving human participants and/or tissue)**<br>• Give the name of the institutional review board or ethics committee that approved the study<br>• Include the approval number and/or a statement indicating approval of this research<br>• Indicate the form of consent obtained (written/oral) or the reason that consent was not obtained (e.g. the data were analyzed anonymously)<br><br>**Animal Research (involving vertebrate animals, embryos or tissues)**<br>• Provide the name of the Institutional Animal Care and Use Committee (IACUC) or other relevant ethics board that reviewed the study protocol, and indicate whether they approved this research or granted a formal waiver of ethical approval<br>• Include an approval number if one was obtained<br>• If the study involved *non-human primates*, add *additional details* about animal welfare and steps taken to ameliorate suffering<br>• If anesthesia, euthanasia, or any kind of animal sacrifice is part of the study, include briefly which substances and/or methods were applied<br><br>**Field Research**<br><br>Include the following details if this study involves the collection of plant, animal, or other materials from a natural setting:<br>• Field permit number<br>• Name of the institution or relevant body that granted permission	
**Data Availability**<br><br>Authors are required to make all data underlying the findings described fully available, without restriction, and from the time of publication. PLOS allows rare exceptions to address legal and ethical concerns. See the PLOS Data Policy and FAQ for detailed information.	Yes - all data are fully available without restriction

A Data Availability Statement describing where the data can be found is required at submission. Your answers to this question constitute the Data Availability Statement and **will be published in the article**, if accepted.<br><br>**Important:** Stating 'data available on request from the author' is not sufficient. If your data are only available upon request, select 'No' for the first question and explain your exceptional situation in the text box.<br><br>Do the authors confirm that all data underlying the findings described in their manuscript are fully available without restriction?	
**Describe where the data may be found in full sentences. If you are copying our sample text, replace any instances of XXX with the appropriate details.**<br><br>• If the data are **held or will be held in a public repository**, include URLs, accession numbers or DOIs. If this information will only be available after acceptance, indicate this by ticking the box below. For example: *All XXX files are available from the XXX database (accession number(s) XXX, XXX.).*<br>• If the data are all contained **within the manuscript and/or Supporting Information files**, enter the following: *All relevant data are within the manuscript and its Supporting Information files.*<br>• If neither of these applies but you are able to provide **details of access elsewhere**, with or without limitations, please do so. For example:<br><br>*Data cannot be shared publicly because of [XXX]. Data are available from the XXX Institutional Data Access / Ethics Committee (contact via XXX) for researchers who meet the criteria for access to confidential data.*<br><br>*The data underlying the results presented in the study are available from (include the name of the third party*	The finding of this study was generated from the data collected and analyzed based on stated methods and materials. The original data supporting this finding are available from the corresponding author on reasonable request.

and contact information or URL).<br>• This text is appropriate if the data are owned by a third party and authors do not have permission to share the data.<br><br>* typeset	
Additional data availability information:	Tick here if the URLs/accession numbers/DOIs will be available only after acceptance of the manuscript for publication so that we can ensure their inclusion before publication.

Powered by Editorial Manager® and ProduXion Manager® from Aries Systems Corporation

Click here to access/download;Manuscript;Original Research Article.docx

The magnitude of under nutrition and associated factors among pregnant women attending Antenatal Care service in Public Hospitals of western Ethiopia.

Authors

Lamessa Tafara[1*], Haile Bikila[2], Ilili Feyisa[2], Markos Desalegn[2], Zalalem Kaba[3].

[1]Lalo Asabi District, Enango town, Western Ethiopia.

[2]Department of Public Health, Institute of Health Sciences, Wollega University, Nekemte, Ethiopia.

[3]Water, Sanitation, Hygiene (WASH), and Neglected Tropical Diseases (NTDs) Program Coordinator, Nekemte, Western Ethiopia.

Corresponding author*:- Lamessa Tafara, Lalo Asabi District Health Office, Oromia, western Ethiopia.

Tel. +251912820505; E-mail: lamesatafara3@gmail.com.

Authors' email Address:

LT: lamesatafara3@gmail.com (**Corresponding author**)

HB: haile.bikila@gmail.com

IF: liafeyisa2@gmail.com

MD: markosdesalegn@gmail.com

ZK: kabazalalem@gmail.com

Abstract

Background: Pregnancy constitutes states of considerable physiological stress, which cause increased nutritional demands. Under nutrition is a worldwide health issue, especially among pregnant women. Malnutrition during pregnancy can result in miscarriages, fetal deaths during pregnancy, pre-term delivery, and maternal mortality for both the mother and her fetus. Therefore, this research was aimed to assess the magnitude of under nutrition and associated factors among pregnant women attending antenatal care services at public hospitals of West Wollega Zone.

Objective: To assess the magnitude of under nutrition and associated factors among pregnant women attending Antenatal Care service in Public Hospitals of western Ethiopia.

Methods: Facility based cross-sectional study was conducted using systematic random sampling techniques. Interviewer administered structured questionnaire was used to collect the data and Mid-upper arm circumference, height and weight were measured to determine the magnitude of under nutrition. Then, the data were analyzed using logistic regression with 95% confidence interval (CI). Besides, the results of data analysis were presented using appropriate descriptive statistics.

Result: The magnitude of under nutrition among pregnant women was found to be 39.2% (95%CI: 35.7%, 42.6%). Rural residence [(AOR=1.97, 95% CI: (1.24, 3.14)], substance use [(AOR: 3.33, 95% CI: (1.63, 6.81)], low dietary diversity of women [(AOR= 7.56, 95% CI: (4.96, 11.51)], mildly food insecure household [(AOR= 4.36, 95% CI: (2.36, 8.79)] ,moderately food insecure household [(AOR= 3.71, 95%CI: (1.54, 8.79), and severely food insecure household [(AOR= 6.96, 95% CI: (3.15, 15.42)] were factors significantly associated with under nutrition.

Conclusion: The magnitude of under nutrition is very high among pregnant women. Rural residency, household food insecurity, dietary diversity and substance use were significant predictors of undernutrition among pregnant women. Therefore, all concerned bodies should made efforts to reduce the risk of under nutrition by reducing substance use and improving household food security there by to increase women's dietary diversity.

Key words: Under nutrition, Pregnancy, Associated factor.

Background

Under nutrition is the result of inadequate intake of food in terms of either quantity or quality, poor utilization of nutrients due to infections or other illnesses, or a combination of these immediate causes [1]. Pregnancy strongly depends on the health and nutritional status of women and a high proportion of pregnant women are affected by poor nutrition which leads them to unhealthy and distress conditions. Under nutrition goes beyond calories and signifies deficiencies in any or all of the following: energy, protein, and/or essential vitamins and minerals [2].

Pregnancy constitutes states of considerable physiological stress, which cause increased nutritional demands. If these demands are in adequate, not only the nutritional status of the subject will affect, but also the course of pregnancy and lactation. Nutrition-related problems form the core of many current issues in women's health, and poor nutrition can have profound effects on reproductive outcomes [3]. Lack of adequate nutrition of good quality and quantity during pregnancy can cause health problems for both the mother and her fetus. Under nutrition is among the most common causes of maternal mortality [1, 4].

The prevalence of undernourishment-the percentage of the population without regular access to adequate calories-has stagnated since 2015, and the number of people who are hungry has actually risen to 822 million from 785 million in 2015[1]. Expectant and nursing mothers, infants and children constitute the most vulnerable segments of a population from the nutritional standpoint. The Global Burden of Disease Study 2013 identified that maternal and child malnutrition causes 1.7 million deaths and 176.9 million DALYs (Disability Adjusted Life Years)[25]. A survey carried out in South India revealed that among poor women whose dietaries during pregnancy provided 1400-1500 calories and about 40 g of protein daily, nearly 20% of pregnancies had terminated in abortions, miscarriages or stillbirths [6].

Maternal under nutrition directly or indirectly causes about 3.5 million deaths of women in developing countries [7]. In developing countries, it has been estimated that poor nutritional status in pregnancy accounts for 14% of fetuses with IUGR (Intra Uterine Growth Retardation), and maternal stunting account for a further 18.5% [8]. If adolescents or women are undernourished during pregnancy, the cycle of maternal malnutrition, fetal growth restriction, child stunting, subsequent lifetime of impaired productivity, and increased maternal and fetal morbidity and mortality are continued [9].

Under nutrition among women in reproductive age is significantly higher in Africa due to chronic energy and/or micronutrient deficiencies especially during pregnancy [10-11]. In developing nations the prevalence of under nutrition among pregnant women ranges from 13% to 38% [12-13]. The situation is worse in Africa that the burden of malnutrition among pregnant women is about 23% [14].

A 2018 WHO (World Health Organization) African region report indicates, nine countries in Africa had a prevalence rates above 15%, this includes Ethiopia in which maternal underweight exceeds 20% [15]. Recent study done among young pregnant mothers in Ethiopia indicates the prevalence of under nutrition is 38% [16]. Individual studies across Ethiopia indicates high rates of under nutrition among pregnant women, ranging from 9.2% to 44, 9% [17-24], making Ethiopia to be one of the countries with highest burden of maternal under nutrition from the world.

Malnutrition is holding back development with unacceptable human consequences [1]. Globally, hunger and malnutrition reduce a Gross Domestic Product (GDP) of a given country by 1.4-2.1 trillion United States Dollar (USD) a year. Similarly, malnutrition costs between 3 and 16% annual GDP of the 54 African countries, and for mentioning Ethiopia loss 16.5% a year [25-26].

Despite efforts made to improve the problem; the progress made in last decade was very low, and currently the burden of under nutrition is continued to be the major public health problem in developing countries including our country Ethiopia [3]. Different studies done across our country tried to show the burden and determinant of under nutrition among pregnant women, in any consideration of the problems of under nutrition, these segments require special consideration. As under nutrition caused by complex interrelated factors the programs and interventions designed to reduce its burden should depend on the reliable and recent information derived from extensive studies targeting this segment of population. Therefore, this study intended to provide valuable information to contribute to existing knowledge on the nutritional status of pregnant women at national and local level.

Methods and materials

Study Design, Area, and Period

An institution-based cross-sectional study design was carried out. This study was conducted in public general hospitals of the Oromia region. The study was conducted in five public hospitals in the zone were the selected study area. This study was conducted in Public Hospitals of western Ethiopia from April 10 to May 10, 2020.

Source and Study population

The source populations were all third-trimester pregnant women who were coming for delivery and antenatal care visits in the selected public general hospitals of the Oromia region. Third-trimester pregnancy women who were coming for antenatal care visits in general public hospitals of the Oromia region western part were selected as the study population.

Inclusion and exclusion criteria

All selected third-trimester pregnant women who were coming for ANC in public general hospitals during the study period were included, whereas pregnancy women with bilateral edema, who were seriously sick and unable to respond to the interview, were excluded from the study.

Sample size and Sampling techniques

Sample size was calculated using double population proportion formula by assuming precision OR (Odd ratio) 1.52 (d) = 5%, confidence level = 95% ($Z\alpha/2$ =1.96), and proportion of under nutrition (*P1* proportion among exposed group) 49.5%, (p2 proportion among exposed group) 39.2%. By, it becomes 768. By considering a 5% non-response rate, 806 pregnant women were taken as a final sample size after using the design effect two.

The sample size was allocated proportional to their average monthly client flow. Systematic sampling was used to select the study units from pregnant women attending ANC. The interval K value was determined for samples at each hospital by dividing the number of units in the population (N) by the desired sample size (n). The first respondent selected by lottery method, and then every second respondent was included until the desired sample size was attained [Figure 1].

Figure 1: Proportional allocation of sample size for Assessment of undernutrition among pregnant women at Public hospitals of West Wollega Zone.

Data collection Procedure

A semi-structured questionnaire was initially prepared in English and then translated into the regional language; Afaan Oromo was used. Afaan Oromo version was again translated back to English to check for any inconsistencies or distortion in the meaning of words. Data were collected using an interviewer-administered, and MUAC

measurement questionnaire was adapted from the literature. Data collection was performed by one BSc Nurse as supervisor and five Midwifery nurses (Diploma) were employed for data collection. To assure the quality of the data properly designed data collection instrument and training of data collectors and supervisors was done, the enumerators and the supervisor were given training for three days on procedures, techniques, ways of collecting the data, and monitoring the procedure especially on anthropometric measurement. Ten percent pretest was done at the, Gida public general hospital to check the consistency of the questioner. The collected data were reviewed and checked for completeness by supervisors and principal investigators each week. MUAC was measured by considering the mothers in Frankfurt plane and sideways to measure the left side, arms hanging loosely at the side with the palm facing inward, taken at marked midpoint of upper left arm, a flexible non stretchable tape were used, and difference between trainee and trainer was 0-5mm after standardization of measurement error calculation before data collection.

Independent and dependent Variable

Nutritional status of pregnant mothers is the outcome variable, and the independent variables were all the sociodemographic characteristics, dietary habit, environmental, maternal obstetrical and gynecology history. A brief description of how some of these variables were measured is as follows.

Dependent variable

The mid-upper arm circumstance values below a cutoff point < 23 cm were considered as under nutrition in this study, whereas for the individual 23 cm and above, was considered normal [13].

Potential confounding factor

Potential confounding variables measured in the study were socio-demographic characteristics, obstetrics and gynecology including the age of mother, marital status, religion, educational background of mothers, women's decision-making autonomy, household income, occupation, ethnicity, number of antenatal care visits, type of pregnancy, maternal previous surgery, malaria, parity, iron and folic acid supplementation, marriage at age, substance use, hemoglobin level, coffee intake, husband's support, difficulty to access food during the last three months, Dietary diversity, household food insecurity, prenatal feeding habits like skipping meals, frequency of meal, habit of eating snack, food avoidance, and food intake and history of low birth weight.

Anthropometric Measurement

The anthropometric measurement mid upper arm circumstance was taken from individual third-trimester pregnant women. Intra-observer and inter-observer variability of anthropometric measurement were assessed on 10 volunteers to reduce technical error of measurement (TEM) at end of training. The measuring instruments were calibrated after each session of measurements. The Supervisor gave close supervision and technical supports, and checked the collected data for completeness, accuracy, and consistency every day and onsite.

Result

Socioeconomic and Demographic Characteristics of Pregnant Women

Out of 806 pregnant initially planned to be included in the study, 780 of them were participated with 96.8% response rate. The mean (±SD) age of respondents was 26±5.32. The median family size of respondents was three persons. Majorities (64.7%) of respondents were Protestant, and about 20% were Orthodox follower. All most all of the participants (97.7% were married. Majority (53.1%) of respondents are urban dwellers while the rest (46.9%) are rural residents. About 24% of respondents completed tertiary education while only 8% had no formal education. Nearly half (48.6%) of participants were Housewife, and only 6.4% of them were daily laborers. Majority (55.4%) of respondent's family have > 1500ETB monthly income [Table 1]. Nearly all 96.7% of participants have latrine near their house, while 83.7% have access to safe water source, and Fifty nine percent of pregnant women have low decision-making autonomy while the rest have high decision-making autonomy [Figure 2].

Figure 2: Environmental and cultural characteristics of participants attending ANC services at Public Hospitals of West Wollega Zone, 2020.

Table 1: Socio-demographic characteristics of participants attending Antenatal care services at Public Hospitals of West Wollega Zone, 2020.

Variables		Frequency	Percent
Religion of respondents	Muslim	72	9.2
	Protestant	505	64.7
	Orthodox	158	20.3
	Others	45	5.8
Marital status of respondents	Single	12	1.5
	Married	762	97.7
	Divorced	6	0.8
Residence	Urban	414	53.1
	Rural	366	46.9
Respondents occupational status	Government employee	151	19.4
	Merchant	118	15.1
	House wife	379	48.6
	Daily laborer	50	6.4
	Student	82	10.5
Couples occupational status	Government employee	182	23.7
	Farmer	253	32.9
	Merchant	134	17.4
	Daily laborer	199	25.9
Age group of respondents	15-24	330	42.3
	25-34	396	50.8
	35-49	54	6.9
Respondents educational status	No formal education	60	7.7
	Primary education	282	36.2

	Secondary education	252	32.3
	Tertiary education	186	23.8
Couples educational status	No formal education	54	7.0
	Primary education	246	32.0
	Secondary education	294	38.3
	Tertiary education	174	22.7
Family size of respondent	≤3	486	62.3
	4-6	240	30.8
	>6	54	6.9
Presence of under five children in the house hold	No	456	58.5
	Yes	324	41.5
Household monthly income	<1000	246	31.5
	1000-1500	102	13.1
	>1500	432	55.4
Note: Others* 7th day Adventists			

Reproductive and Health Care characteristics of the respondents

A mean (±SD of age at first marriage, number of pregnancy, and gestational age of respondents were 19(±2.14) years, 2(±1.16) pregnancy, and 30(±5.1) weeks respectively. About three fourth of participants were married at age of 18 years and above. Majority (63.8%) of respondents were at their third trimesters of pregnancy, about two third 68.5 of them were multigravida. Seven hundred and two of respondents (90%) said their current pregnancy was intended. About 78% of respondents were used any type of contraceptive before current pregnancy. One hundred fifty six (20%) of respondents reported history of pregnancy related complication, 8.5% reported history of current illness, and 3.8% reported history of chronic illness, while only 6.3% of them have history of substance use[Table 2].

Table 2: Reproductive and medical characteristics of participants attending Antenatal care services at Public Hospitals of West Wollega Zone, 2020 (N=780).

**Variables**		**Frequency**	**Percent**
Age at first marriage of respondents (N=774)	<18 years	132	17.1
	≥ 18 years	642	82.9
Trimesters of pregnancy	Second	282	36.2
	Third	498	63.8
Number of pregnancy	Prim gravida	246	31.5
	Multigravida	534	68.5
Number of birth (N=528)	Null para	276	52.3
	Multipara	252	47.7
Previous birth interval (N=252)	<2 years	30	11.9
	2 to 4 years	150	59.5
	>= 4 years	72	28.6
Intention of current pregnancy	No	78	10.0
	Yes	702	90.0
Number of antenatal care visit	First visit	210	26.9
	Second visit	216	27.7
	Third visit	210	26.9
	Fourth visit	144	18.5
Previous contraceptive use	No	174	22.3
	Yes	606	77.7
Nutritional advice during pregnancy (N=570)	No	276	48.4
	Yes	294	51.6
Use of iron and folic acid supplementation (N=570)	No	36	6.3

	Yes	534	93.7
Deworming (N=570)	No	473	83.0
	Yes	97	17.0
History of pregnancy complication	No	624	80.0
	Yes	156	20.0
History of current illness	No	714	91.5
	Yes	66	8.5
History of frequent illness	No	708	90.8
	Yes	72	9.2
History of chronic illness	No	750	96.2
	Yes	30	3.8
Substance use	No	731	93.7
	Yes	49	6.3

Dietary characteristics of the respondents

Two hundred and thirty six (30.3%) of participants respond as consuming meals less than three times a day while majority of respondents (62.3%) of them said not increased their meals since pregnancy. Nearly half (49.2%) of pregnant women reported no habit of eating snack. Only 14.6% of participants have habit of fasting, while 18.5% have food avoidance and 8.5% have habit of skipping meal during current pregnancy.

More than three forth (80%) of pregnant women have poor prenatal feeding habit, 40% of them consumed low dietary diversity. From total participants, 600(76.9%) were from food secure, 10.8% were from mildly food insecure, 5.4% were from moderately food insecure, and 6.9% were from severely food insecure household [Table 3]. All most all (97.7%) of the participants adequately consume cereals, more than three forth (80.1%) adequately consume legumes, and more than half (58.3%) adequately consume dark green leafy vegetables. Less than half of

respondents adequately consume the rest of food group listed in the table below, except milk and its products, which no participants have adequately consumed during last four weeks before the study [Table 4].

Table 3: Prenatal feeding habits of participants attending Antenatal care services at Public Hospitals of West Wollega Zone, 2020.

**Variables**		**Frequency**	**Percent**
Frequency of meals in a day	<3	236	30.3
	≥3	544	69.7
Increased frequency of meals	No	486	62.3
	Yes	294	37.7
Habit of eating snack	No	384	49.2
	Yes	396	50.8
Habit of fasting	No	666	85.4
	Yes	114	14.6
Food avoidance	No	636	81.5
	Yes	144	18.5
Habit of skipping meal	No	714	91.5
	Yes	66	8.5
Prenatal feeding habits of respondents	Poor	630	80.8
	Good	150	19.2
Dietary diversity of woman	Low	312	40.0
	High	468	60.0
Household food insecurity status	Food secure	600	76.9
	Mild	84	10.8
	Moderate	42	5.4
	Severe	54	6.9

Table 4:-Consumption of common food groups among participants attending ANC services at Public Hospitals of West Wollega Zone, 2020.

Variable	Category	Frequency	Percent
Cereals intake	Inadequate	18	2.3
	Adequate	762	97.7
Legumes intake	Inadequate	155	19.9
	Adequate	625	80.1
Dark green leafy vegetables intake	Inadequate	325	41.7
	Adequate	455	58.3
Yellow orange vegetables intake	Inadequate	522	66.9
	Adequate	258	33.1
White roots and tubers intake	Inadequate	532	68.2
	Adequate	248	31.8
Flesh meats intake	Inadequate	696	89.2
	Adequate	84	10.8
Milk and milk products intake	Inadequate	768	98.5
	Adequate	12	1.5
Eggs intake	Inadequate	672	86.2
	Adequate	108	13.8
Oils and fats intake	Inadequate	522	66.9
	Adequate	258	33.1

Nutritional status of respondents

The mean (±SD) MUAC of pregnant women studied was 24.24 (±2.7) cm. The magnitude of under nutrition (MUAC <23cm) was 39.2%, (95%CI: 35.7%, 42.6%) [Figure 3].

Figure 3: Nutritional status of participants attending ANC services at Public Hospitals of West Wollega Zone.

Results of Logistic Regression Analysis

Under nutrition was taken as a dependent variable and compared against each independent variable for association. Bivariable logistic regression was done to identify factors associated with nutritional status of pregnant women. Accordingly, household food insecurity, low dietary diversity, poor prenatal feeding habits, number of pregnancy, trimesters of pregnancy, age at first marriage less than 18 years, family size ≥6, substance use, chronic illness, rural residence , not eating snack, not increase frequency of meal shows significant association with under nutrition crudely at 25%[Table 5, 6 and 7].

Table 5: Bivariate analysis of Socio demographic factors associated with undernutrition among pregnant women attending ANC at West Wollega public Hospitals, 2020

**Associated Factors**		**Undernutrition (MUAC<23)**		**P-Value**	**COR**	**95%C.I for COR**	
		**Yes Count (%)**	**No Count (%)**			**Lower**	**Upper**
Age group of respondents	15-24	138(41.8)	192(58.2)	0.717	0.89	0.50	1.60
	25-34	144(36.4)	252(63.6)	0.251	0.71	0.40	1.27
	35-49	24(44.4)	30(55.60)		1		
Respondents residence	rural	198(54.1)	168(45.9)	0.000	3.34	2.47	4.51*
	urban	108(26.1)	306(73.9)		1		
Household monthly income	<1000	112(47.5)	124(52.5)	0.263	1.29	0.79	5.25
	1000-1500	46(45.1)	56(54.9)	0.156	1.17	0.54	3.98
	>1500	178(41.2)	254(58.8)		1		
Family size of respondent	>=6	30(55.6)	24(44.4)	0.039	1.82	1.03	3.20*
	4-5	78(32.5)	162(67.5)	0.032	0.70	0.51	0.97
	<=3	198(40.7)	288(59.3)		1		
Presence of under five children	Yes	138(42.6)	186(57.4)	0.105	0.79	0.59	1.05
	No	168(36.8)	288(63.2)		1		

Sources of drinking water	Unsafe	48(80)	12(20)	0.479	1.15	0.78	1.68
	Safe	258(35.8)	462(64.20)		1		
No latrine	Yes	12(50)	12(50)	0.276	1.57	0.70	3.54
	No	294(38.9)	462(61.1)		1		
Decision making autonomy	Low	186(40.3)	276(59.7)	0.478	1.11	0.83	1.49
	High	120(37.7)	198(62.3)		1		
Substance use	Yes	32(65)	17(34.7)	0.000	3.14	1.71	5.76*
	No	274(37.5)	45762.5)		1		
Respondents educational status	No formal education	66(55)	54(45)	0.528	1.21	0.67	2.20
	Primary education	162(52.9)	144(47.1)	0.127	1.35	0.92	1.97
	Secondary education	66(28.9)	162(71.1)	0.576	1.12	0.75	1.66
	Tertiary education	12(9.5)	114(90.5)		1		
Couples educational status	No formal education	72(60)	48(40)	0.393	1.31	0.71	2.43
	Primary education	108(50)	108(50)	0.425	0.85	0.57	1.27
	Secondary education	9638.1)	156(61.9)	0.141	1.33	0.91	1.96
	Tertiary education	30(16.7)	150(83.3)		1		
Respondents occupational status	farmer	66(45.8)	78(54.2)	0.258	1.33	0.81	2.17
	merchant	18(25)	54(75)	0.867	1.03	0.70	1.53
	house wife	198(51.6)	186(48.4)	0.651	1.16	0.60	2.24
	daily laborer	18(33.3)	36(66.7)	0.099	1.58	0.92	2.73
	government employee	6(6.7)	84(93.3)		1		
Couples occupational status	farmer	192(56.1)	150(43.9)	0.094	1.40	0.94	2.06
	merchant	18(15)	102(85)	0.551	1.15	0.73	1.82
	daily laborer	72(52.2)	66(47.8)	0.774	1.06	0.70	1.61
	government employee	24(14.8)	138(85.2)		1		
Note: * = statistically significant at p-value < 0.25, 1: reference category, COR: Crude odds ratio, 95%CI: 95 percent confidence interval							

Table 6: Bivariate analysis of Reproductive and medical factors associated with undernutrition among participants attending ANC services at West Wollega public Hospitals, 2020

**Associated Factors**		**Undernutrition (MUAC<23)**		**P-Value**	**COR**	**95% CI for COR**	
		**Yes, Count (%)**	**No. Count (%)**			**Lower**	**Upper**
Number of pregnancy	multigravida	228(42.7)	306(57.3)	0.004	1.61	1.17	2.21*
	prim gravida	78(31.7)	168(68.3)		1		
Trimesters of pregnancy	3rd trimester	174(34.9)	324(65.1)	0.001	0.61	0.45	0.82*
	2nd trimester	132(46.8)	150(53.2)		1		
Age at first marriage	< 18 years	96(72.7)	36(27.3)	0.000	5.49	3.62	8.32*
	>= 18 years	210(32.7)	432(67.3)		1		
History of illness in current pregnancy	Yes	30(45.5)	36(54.5)	0.280	1.32	0.80	2.20
	No	276(38.7)	438(61.3)		1		
History of chronic illness	Yes	18(60)	12(40)	0.021	2.41	1.14	5.06*
	No	288(38.4)	262(61.6)		1		
History of pregnancy complication	Yes	60(38.5)	96(61.5)	0.826	0.96	0.67	1.38
	No	246(39.40)	378(60.60)		1		
No previous contraceptive use	Yes	72(41.40)	102(58.60)	0.510	1.12	0.80	1.58
	No	234(38.60)	372(61.40)		1		
Number of antenatal care visit	first visit	84(40)	126(60)	0.276	0.79	0.51	1.21
	second visit	66(30.60)	150(69.4)	0.003	0.52	0.34	0.81
	third visit	90(42.9)	120(57.1)	0.580	0.89	0.58	1.36
	fourth visit	66(45.8)	78(54.2)		1		
Non intended pregnancy	Yes	36(46.2)	42(53,8)	0.188	1.37	0.86	2.20
	No	270(38.5)	432(61.5)		1		
Note: * = statistically significant at p-value < 0.25, 1: reference category, COR: Crude odds ratio, 95%CI: 95 percent confidence interval							

Table 7:-Bivariate analysis of dietary factors with nutritional status of pregnant women attending antenatal care clinics in public hospitals of west Wollega Zone, 2020.

**Associated Factors**		**Undernutrition (MUAC<23)**		**P-Value**	**COR**	**95% CI for COR**	
		**Yes, Count (%)**	**No, Count (%)**			**Lower**	**Upper**
Less than three meals in a day	Yes	95(40.3)	141(59.7)	0.700	1.06	0.78	1.45
	No	211(38.8)	333(61.2)		1		
No habit of eating snack	Yes	186(48.4)	198(51.6)	0.000	2.16	1.61	2.89*
	No	120(30.3)	276(69.7)		1		
Not increased frequency of meals	Yes	240(49.4)	246(50.6)	0.000	3.37	2.43	4.67*
	No	66(22.4)	228(77.6)		1		
Food avoidance during pregnancy	Yes	54(37.5)	90(62.5)	0.638	0.91	0.63	1.33
	No	252(39.60)	384(60.4)		1		
Habit of fasting while pregnant	Yes	54(47.4)	60(52.6)	0.055	1.48	0.99	2.20
	No	252(37.8)	414(62.2)		1		
Habit of skipping meal	Yes	54(81.8)	12(18.2)	0.000	8.25	4.33	15.71*
	No	252(35.3)	462(64.7)		1		
Prenatal feeding habits	Poor	187(29.7)	443(70.3)	0.236	0.84	0.25	1.67
	Good	50(33.3)	150(66.7)		1		
Household food insecurity status	Severe	42(77.9)	12(22.2)	0.000	8.57	4.41	16.67*
	Moderate	30(71.4)	12(28.8)	0.000	6.12	3.06	12.23*
	Mild	60(71.4)	24(28.6)	0.000	6.12	3.69	10.14*
	Food secure	174(29)	426(71)		1		
Dietary diversity of woman	Low	222(71.2)	90(28.8)	0.000	11.28	8.03	15.84*
	High	84(17.9)	384(82.0)		1		
Dark green leafy vegetables intake	inadequate	121(37.2)	204(62.8)	0.334	0.87	0.65	1.16

	adequate	185(40.7)	270(59.3)		1		
Yellow orange vegetables intake	inadequate	216(41.4)	306(58.6)	0.081	1.32	0.97	1.80
	adequate	90(34.9)	168(65.1)		1.0		
White roots and tubers intake	inadequate	207(38.9)	325(61.1)	0.788	0.96	0.70	1.31
	adequate	99(39.9)	149(60.1)		1		
Flesh meats intake	inadequate	270(38.8)	426(61.2)	0.472	0.85	0.53	1.34
	adequate	36(42.9)	48(57.1)		1		
Eggs intake	inadequate	265(39.4)	407(60.6)	0.771	1.06	0.70	1.62
	adequate	41(38)	67(62.0)		1		
Oils and fats intake	inadequate	202(38.7)	320(61.3)	0.664	0.94	0.69	1.27
	adequate	104(40.3)	154(59.7)		1		
Note: * = statistically significant at p-value < 0.25, 1: reference category, COR: Crude odds ratio, 95%CI: 95 percent confidence interval							

Variables associated with adjusted analysis: pregnant women, accordingly, household food insecurity, low dietary diversity, substance use and residence were identified as independent predictors of under nutrition among pregnant women. The odds of under nutrition were four times [AOR=4.36, 95%CI:(2.36, ,8.79)] more among mildly food insecure household, and nearly four times [AOR= 3.71, 95%CI: 1.54 ,8.61),among moderately food insecure households, and six times [AOR = 6.96, 95% CI: (3.15,15.42)] among severely food insecure household) compared with their food secure counterparts. Pregnant women with low dietary diversity had seven times [AOR = 7.56, 95% CI :(4.96, 11.51)] increased odds of under nutrition than those with high dietary diversity status.

Moreover, the odds of under nutrition was three times [AOR = 3.33, 95%CI: 1.63, 6.81)] among substance users - than their counter- parts. Rural pregnant women had nearly three times [AOR =2.68, 95%CI: 1.77, 4.06)] increased odds of under nutrition than urban women [Table 8].

Table 8: Multivariate analysis of factors associated with under nutrition among pregnant women attending ANC services at public hospitals of west Wollega Zone.

**Associated factors**		**Undernutrition(MUAC<23cm)**		**Bivariate analysis**			**Multivariable analysis**		
				**COR**	**95% C.I COR**		**AOR**	**95% C.I AOR**	
		**Yes**	**No**		**Lower**	**Upper**		**Lower**	**Upper**
Household food insecurity status	Severe	42(77.9%)	12(22.2%)	8.57	4.41	16.67	6.96	3.15	15.42**
	Moderate	30(71.4%)	12(28.8%)	6.12	3.06	12.23	3.71	1.54	8.96**
	Mildly	60(71.4%)	24(28.6%)	6.12	3.69	10.14	4.55	2.36	8.79**
	Food secure	174(29%)	426(71%)	1			1		
Dietary diversity of woman	Low	222(71.2%)	90(28.8%)	11.28	8.03	15.85	7.56	4.96	11.51**
	High	84(17.9%)	(384)82.0%	1			1		
Prenatal feeding habits	Poor	343(54.4%)	287(45.6%)	5.77	3.48	9.57	1.81	0.86	3.83
	Good	131(87.3%)	19(12.7%)	1			1		
Number of pregnancy	Multigravida	228(42.7%)	306(57.3%)	1.61	1.17	2.21	0.98	0.60	1.60
	Prim gravida	78(31.7%)	168(68.3%)	1			1		
Trimesters of pregnancy	Third trimester	174(34.9%)	324(65.1%)	0.61	0.45	0.82	0.78	0.52	1.17
	Second trimester	132(46.8%)	150(53.2%)	1			1		
Age at first marriage	< 18 years	96(72.7%)	36(27.3%)	5.49	3.62	8.32	1.62	0.93	2.82
	>= 18 years	210(32.7%)	432(67.3%)	1			1		
Family size	>=6	30(55.6%)	24(44.4%)	1.82	1.03	3.2	0.47	0.22	1.00
	4_5	78(32.5%)	162(67.5%)	0.7	0.51	0.97	0.77	0.48	1.23
	<=3	198(40.7%)	288(59.3%)	1			1		
Substance use	Yes	32(65%)	17(34.7%)	3.14	1.71	5.76	3.33	1.62	6.81**

	No	274(37.5%)	457(62.5%)	1			1		
History of chronic illness	Yes	18(60%)	12(40%)	2.41	1.14	5.07	2.83	0.98	8.12
	No	288(38.4%)	262(61.6%)	1			1		
Respondents residence	Rural	198(54.1%)	168(45.9%)	3.34	2.47	4.51	2.68	1.77	4.06**
	Urban	108(26.1%)	306(73.9%)	1			1		
No habit of eating snack	Yes	186(48.4%)	198(51.6%)	2.16	1.61	2.9	1.07	0.67	1.69
	No	120(30.3%)	276(69.7%)	1			1		
Not increased frequency of meals	Yes	240(49.4%)	246(50.6%)	3.37	2.43	4.67	1.21	0.70	2.07
	No	66(22.4%)	228(77.6%)	1			1		
Note: ** indicates statistically significant at P-value < 0.05, 1 indicates reference, COR = crude odd ratio, AOR = adjusted odd ratio									

Discussion

The current study tried to reveal the magnitude and factors associated with undernutrition among pregnant women in West Wollega Zone, Western part of Ethiopia. Accordingly, nearly forty percent (39.2%) of participants were undernourished and factors associated with their nutritional status were residency, substance use, household food insecurity and low dietary diversity of women.

The global estimate of maternal malnutrition during pregnancy appears to be decreasing in almost all regions of the globe except in Africa where the number of pregnant mothers with malnutrition has been increasing steadily over time, this shows that the result of this study is relevance to the current status of undernutrition in Africa, and Ethiopia in particular[14]. The finding of this study was almost consistent with the study conducted in the South western part of our country which shows 44.9% [24] and study conducted in Southern part of Ethiopia which was 35.5% [27].

On the other hand, this study result was lower than study done in Kunama population, Tigray, northern Ethiopia, which was observed to be 47.9%, this may be due to that in the above study the proportion of Food insecurity is higher than the present study which could increase the risk of undernutrition [28]. But lower prevalence of undernutrition was reported among studies conducted in Gambella Town (28.6%), Alamata General Hospital

(23.2%), rural communities in Haramaya district (19.06%), and Dessie town (19.5%) [17-18,21,29]. The discrepancies between the findings may be due to geographical variation between the studies or due to the variation in the cut off point for MUAC measurement that the studies above uses lower cut off points than the present study, which could under estimate the prevalence of undernutrition.

According to this study, rural residents were three times more likely to have undernutrition than urban ones. It is true that place of residence usually determines people's life-styles, income, and most notably their health conditions and nutrition [30]. This finding is in line with the finding of systematic review done in Africa, the study done in University of Gondor Hospital, and the study done in Boricha Woreda, Sidama Zone which indicates rural pregnant women were more likely to develop undernutrition than urban ones(13,21,50). But this finding may not be true in developed world, in that one study in USA shows there is no significant association between nutritional status and residency [31].

Another factor that shows independent prediction with under nutrition in this study was substance use. Accordingly, pregnant women who were using substance show three times more risk of undernutrition than those who were not using substance. Women with substance use disorders frequently experience inadequate prenatal care, chronic medical problems, poverty, and domestic violence that may increase vulnerability to poor nutrition [32]. The finding of study in South Western Ethiopia similarly shows that women with history of substance use were two times more at risk of under nutrition than who were not using any substance [24]. Study done in Rural Eastern Ethiopia also reported the risk of undernutrition is 29% more among substance users [31].

In this study, pregnant women who were consuming low dietary diversity were more than seven times more likely be undernourished than those who were consuming high dietary diversity. The finding of this study is consistent with the study done in Dessie town which shows women who had low dietary diversity were nearly six times more likely to contract undernutrition than the other ones[17]. The study done in Gambella town also shows that pregnant women who had low dietary diversity score were two times more at risk of undernutrition than their counter ones[23]. Survey done in Iran, and a study conducted in Kenya were also among other studies that shows similar finding with this study [33-34]. In contrast to this study, dietary diversity was not show any significant correlation

with maternal anthropometry in rural Cambodia this may be due to that very proportion of women shows prevalence of low dietary diversity in the study done in rural Cambodia[35].

In this study, respondents who were from food insecure household shows more at risk of malnutrition than those from food secure households. Our finding of an increased prevalence of maternal undernutrition in food insecure households may reflect inequitable intra-household food allocation whereby the nutritional needs of the child and/or other members of the household are prioritized over those of the mother. As evidenced from 2019 FAO food insecurity report that, household food insecurity was found to be associated with more than one form of malnutrition [30]. Similar finding was reported from study done in Gumay district, study conducted in Gambella Town, and study done in Kunama population in Tigray region [23-24, 28]. Study done in Nepal also indicates significant relationship between food adequacy and low nutritional status of pregnant women [36]. In contrast to this, increased maternal anthropometry was observed among women from mildly food insecure households in USA, Brazil and Lebanon, this discrepancy may be due to that these studies were from developed world with different sociodemographic characteristics, and they have different method of assessment when compared to present study [37-39].

Educational status of respondents does not show significant association with under nutrition in current study. But this finding contradicts the finding of the study done in Shashamene district, southern part of Ethiopia which shows literate women were 70% reduction in risk of undernutrition than those who had no formal education [40]. Study done in University of Gondor Hospital also shows the risk of undernutrition was nearly three times among pregnant women with no formal education [22]. The study done in rural Nepal also reported there was a significant relationship between the educational level of the women and the general nutritional status [34]. The reason might be because; food and related factors that have an effect on nutritional status of women are under the control of the household head, even though the women had higher education.

Limitations of the study

- Even though this study tried to cover several variables, it does not include variables that need laboratory investigations such as intestinal parasites, and malaria infection.

- Dietary intake of respondents was measured only on occasional time, and that may not show the seasonal variability on availability of food.
- This study used anthropometric measure to assess nutritional status of pregnant mothers and the effect of technical error was not ruled out that may affect the reliability of result.
- As it is institutional based study, the finding of this study does not fully indicate the characteristics of respondents at community level which means it is not generalized beyond study population.

Conclusions and recommendations

Conclusion

This study revealed that, nearly forty percent of pregnant women were undernourished showing high prevalence that needs priority attention intervention. The factors that significantly associated with undernutrition were respondent's residency, substance use, low dietary diversity, and household food insecurity status. The remaining factors studied were not showing any significant association with undernutrition.

Recommendations

Based on the finding of this study the following recommendations were forwarded.

- Governments should adopt coherent policies, which foster cross-sectoral cooperation and strategies to avert the problem of undernutrition among pregnant women.
- Policy makers and implementers should make programs that improve food security status at household level to increase access to high nutritious food and variety of foods among poor.
- Essential investments must be made into nutrition-sensitive programme in other areas such as agriculture, education, water and social protection.
- Agricultural sector should implement nutrition sensitive interventions such as increased productivity and dietary diversifications.
- Health workers should disseminate useful information about the harmful effect of using substance during pregnancy at health institution and community level in order to improve the dietary habits of pregnant women especially for rural residents.

- Health workers should also advice pregnant women about the benefits of dietary diversity during pregnancy.

Acknowledgements

We are grateful to officials from Zonal health office and public Hospitals of West Wollega zone, and the health workers of each facility for their valuable contribution during the study. We also extend our thanks to data collectors, respondents, and supervisors for their cooperation during the study.

Lists of acronyms

- ANC: Antenatal Care
- AOR: Adjusted Odds Ratio
- COR: Crude Odds Ratio
- DALYs: Disability Adjusted Life Years
- FAO: Food And Agricultural Organization
- FFQ: Food Frequency Questionnaire
- GDP: Gross Domestic Product
- GHI: Global Hunger Index
- HFIAS: Household Food Insecurity Access Scale
- MCH: Maternal And Child Health
- MDDW: Minimum Dietary Diversity Of Women
- MUAC: Mid Upper Arm Circumference
- SDGs: Sustainable Development Goals
- UN: United Nations
- UNICEF: United Nations International Children's Emergency Fund
- USA: United States of America
- WHO: World Health Organization

Declarations

Ethics approval and consent to participate

The study protocol was approved and permission was obtained from Wollega University. An official letter of co-operation was written to the selected Public Hospitals of West Wollega Zone Administration. Information on the studies, including purpose and procedures was given for participants. Written or verbal consent was obtained from each participant. In order to protect confidentiality, names or identifications were not included on the written questionnaires. Identification of the respondents was only through numerical codes.

Consent for publication

'Not applicable'

Availability of data and materials

The finding of this study was generated from the data collected and analyzed based on stated methods and materials. The original data supporting this finding are available from the corresponding author on reasonable request.

Competing interests

"The authors declare that they have no competing interests"

Funding

'Not applicable'

Authors' contributions

LT participated in the design of the study, performed the data collection and the statistical analysis and served as the corresponding author of the manuscript. **HB, IF, ZK** and **MD** supervised the study, ensured quality of the data, assisted in the analysis and interpretation of the data. All authors read and approved the manuscript.

Authors' information

- **LF** is Public Health Specialist at Enango health center, Lalo Asabi district, Oromia, Western Ethiopia.
- **HB** is Lecturer at Wollega University department of Public Health, Institute of Health Science, Nekemte, Western Ethiopia.
- **IF** is Lecturer at Wollega University department of Public Health, Institute of Health Science, Nekemte,

Western Ethiopia.

- **MD** is Lecturer at Wollega University department of Public Health, Institute of Health Science, Nekemte, Western Ethiopia.
- **ZK** is Water, Sanitation, Hygiene (WASH) and Neglected Tropical Diseases (NTDs) Program Coordinator, Nekemte town, Western Ethiopia.

Reference

1. K. von Grebmer, J. Bernstein, R. Mukerji, F. Patterson, M. Wiemers, R. Ní Chéilleachair, C. Foley, S. Gitter, K. Ekstrom and HF. 2019 Global hunger index: The challenge of Hunger and climate change. Bonn: Welthungerhilfe; and Dublin:; 2019. Available at www.globalhungerindex.org: Accessed on 23 December 2019.
2. Development Initiatives. Global Nutrition Report 2018: Shining a light to spur action on nutrition. Bristol, UK; 2018. Available at www.ifpri.org. Accessed on 23 December 2019.
3. Safaa Salem, Eshra D SN. Effect of malnutrition during pregnancy on pregnancy outcomes. 18th Int Conf Nurs Heal care. 2016;5(10):4172.
4. Tang AM, Chung M, Dong K, Terrin N, Edmonds A, Chetty T, et al. Determining a Global Mid-Upper Arm Circumference Cutoff to Assess Malnutrition in Pregnant Women. FANTA. Washuington DC; 2016. Available at www.fantaproject.org Accessed on 23 December 2019.
5. Black RE, Allen LH, Bhutta ZA, Caulfield LE, Onis M De, Ezzati M, et al. Maternal and child undernutrition : global and regional exposures and health consequences. Lancet Ser. 2008;371(January 17):5–22.
6. Gopalan C, Ph D. Effect of Nutrition on Pregnancy and Lactation: Paper submitted to the Joint FAO/WHO Expert Com- mittee on Nutrition. 1962;26:203–11.
7. ECA A NW. The Cost of Hunger in Africa : Social and Economic Impact of Child Undernutrition in Egypt , Ethiopia , Swaziland and Uganda Background paper. Abuja, Nigeria; 2014.
8. Blössner M, Onis M De, Prüss-üstün A, Campbell-lendrum D, Corvalán C, Woodward A. Malnutrition Quantifying the health impact at national and local levels. World Heal Organ. 2005;(12):1–51.

9. R. Black, C. Victora, S. Walker et al., "Maternal and child under nutrition and overweight in low-income and middle-income countries,"; *e Lancet*, vol. 382, no. 9890, pp. 427–451, 2013).
10. Adebowale SA, Adepoju OT, Okareh OT FF. Social epidemiology of Adverse nutritional Outcome among women in nigeria. Pakista J Nutr. 2011;10(9):888-98.
11. Lartey A. Maternal and child nutrition in Sub-Saharan Africa : challenges and interventions. Proc Nutr Soc. 2020;67(2008):105-8.
12. Ravishankar AK, Ramachandran S SA. Prevalence of chronic energy deficiency and its consequence on children's nutritional status among the marginalized group in India. 2013;43–4.
13. Devgun P, Mahajan SL, Gill KP. Prevalence of chronic energy deficiency and socio demographic profile of women in slums of Amritsar city , Punjab , India. Int J Res Heal Sci [Internet]. 2014;(2):527–32. Available from: http://www.ijrhs.com/issues.php?val=Volume2&iss=Issue2%0AHarvard
14. Demelash H, Id D, Dadi AF. Burden and determinants of malnutrition among pregnant women in Africa : A systematic review and meta-analysis. . PLoS ONE 14(9) e0221712. 2019;14(19):1-19.
15. World Health Organization. Nutrition in the WHO African Region. Brazzaville: World Health Organization; 2017. 1–85. available at http://apps.who.int/iris. accessed on 23 December 2019
16. Workicho A, Kolsteren P. Burden and determinants of undernutrition among young pregnant women in Ethiopia. Matern child Nutr. 2018;(November):1–9.
17. Diddana TZ. Factors associated with dietary practice and nutritional status of pregnant women in Dessie town , northeastern Ethiopia : a community-based cross-sectional study. BMC Pregnancy Childbirth. 2019;19:1–10.
18. Kedir H, Berhane Y, Worku A. Magnitude and determinants of malnutrition among pregnant women in eastern Ethiopia : evidence from rural , community-based setting. Matern Child Nutr. 2016;12:51–63.
19. Kuche D, Singh P, Moges D, Belachew T. Nutritional Status and Associated Factors among Pregnant Women in Wondo Genet District, Southern Ethiopia. J Food Sci Eng. 2015;5:85–94.
20. Dadi AF DH. Undernutrition and its associated factors among pregnant mothers in Gondar town, Northwest Ethiopia. PLoS One. 2019;14(4).
21. Dagne S. Undernutrition and associated factors among pregnant women attending ANC follow up in Alamata general hospital , Northern Region , Ethiopia ,. J Nutr Heal Food Eng. 2019;9(3):70–8.

22. Kumera G, Gedle D, Alebel A, Feyera F, Eshetie S. Undernutrition and its association with socio-demographic , anemia and intestinal parasitic infection among pregnant women attending antenatal care at the University of Gondar Hospital , Northwest Ethiopia. BMC Nutr. 2018;4:1–10.

23. Gebrehiwot TT, Gemeda DH. Household Food Insecurity , Low Dietary Diversity , and Early Marriage Were Predictors for Undernutrition among Pregnant Women Residing in Gambella , Ethiopia. Hindawi. 2018;2018:10.

24. Shiferaw A H. Acute Under Nutrition and Associated Factors among Pregnant Women in Gumay District, Jimma Zone, South West Ethiopia. J Women ' s Heal Care. 2019;8(2):1–12.

25. Forouzanfar MH, Alexander L, Anderson HR, Bachman VF, Biryukov S BM. Global , regional , and national comparative risk assessment of 79 behavioural , environmental and occupational , and metabolic risks or clusters of risks in 188 countries , 1990 – 2013 : a systematic analysis for the Global Burden of Disease Study 2013. Lancet 2015. 2015;386(10010):2287–323.

26. WHO. Nutrition of women in the preconception period, during pregnancy and the breastfeeding period. New york; 2011.

27. Mathewos M. Eskindir L. Nutritional status and associated factors among pregnant women in Boricha Woreda, Sidama Zone, Southern Ethiopia, 2013: Institute. Hawassa Univ Coll Med Heal Sci Amare Werku Addis Cont. 2013.

28. Abraham S, Miruts G, Shumye A. Magnitude of chronic energy deficiency and its associated factors among women of reproductive age in the Kunama population ,. BMC Nutr. 2015;1(12):1-9.

29. Tafese Z, Kebebu A. A systematic review of maternal feeding practice and its outcome in developing countries. Res J Food Sci Nutr. 2017;2:9-14.

30. FAO, IFAD, UNICEF W and W. Food security and nutrition in the world. safeguarding against economic slowdowns and downturns. Rome, Italy; 2019. 1–212 p.

31. Hill JL, You W, Zoellner JM. Disparities in obesity among rural and urban residents in a health disparate region. BMC Public Health [Internet]. 2014;14(1051):1–8. Available from: www.biomedcentral.com/1471-2458/14/1051%0ARESEARCH

32. Forray A, Haven N. Substance use during pregnancy open peer review. F1000research Rev. 2016;5(May):1-9.

33. Vakili M, Abedi P, Sharifi M, Hosseini M. Dietary Diversity and Its Related Factors among Adolescents : A Survey in Ahvaz-Iran. Glob J Health Sci. 2013;5(2):181–6.

34. Lillian M. Dietary Diversity and Nutritional Status of Pregnant Women Aged 15-49 YearS Attending Kapenguria District Hospital West Pokot County , Kenya. 2013;1:49.

35. Mcdonald CM, Mclean J, Kroeun H, Talukder A, Lynd LD, Green TJ. Household food insecurity and dietary diversity as correlates of maternal and child undernutrition in rural Cambodia. Eur J Clin Nutr [Internet]. 2015;69(2):242–6. Available from: http://dx.doi.org/10.1038/ejcn.2014.161

36. Acharya SR, Bhatta J, Timilsina DP. Factors associated with nutritional status of women of reproductive age group in rural , Nepal. Asian Pacific J Heal Sci |. 2017;4(4):19-24.

37. Jomaa L, Naja F, Cheaib R, Hwalla N. Household food insecurity is associated with a higher burden of obesity and risk of dietary inadequacies among mothers in. BMC Public Health. 2017;17(567):1–14.

38. Peterson K, Peterson K. Household Food Insecurity And Obesity Risk In An Urban Slum In Brazil By. EliScholar – A Digit Platf Sch Publ Yale Univ. 2014;(January):1–47.

39. Olson CM. Symposium : Advances in Measuring Food Insecurity and Hunger in the U . S . and Hunger 1. Am Soc Nutr Sci. 1999;521-4.

40. Belete Y, Negga B, Firehiwot M. Under Nutrition and Associated Factors among Adolescent Pregnant Women in Shashemenne District , West Arsi Zone , Ethiopia : A Community- based Study. J Nutr Food Sci. 2016;6(1):1-7.

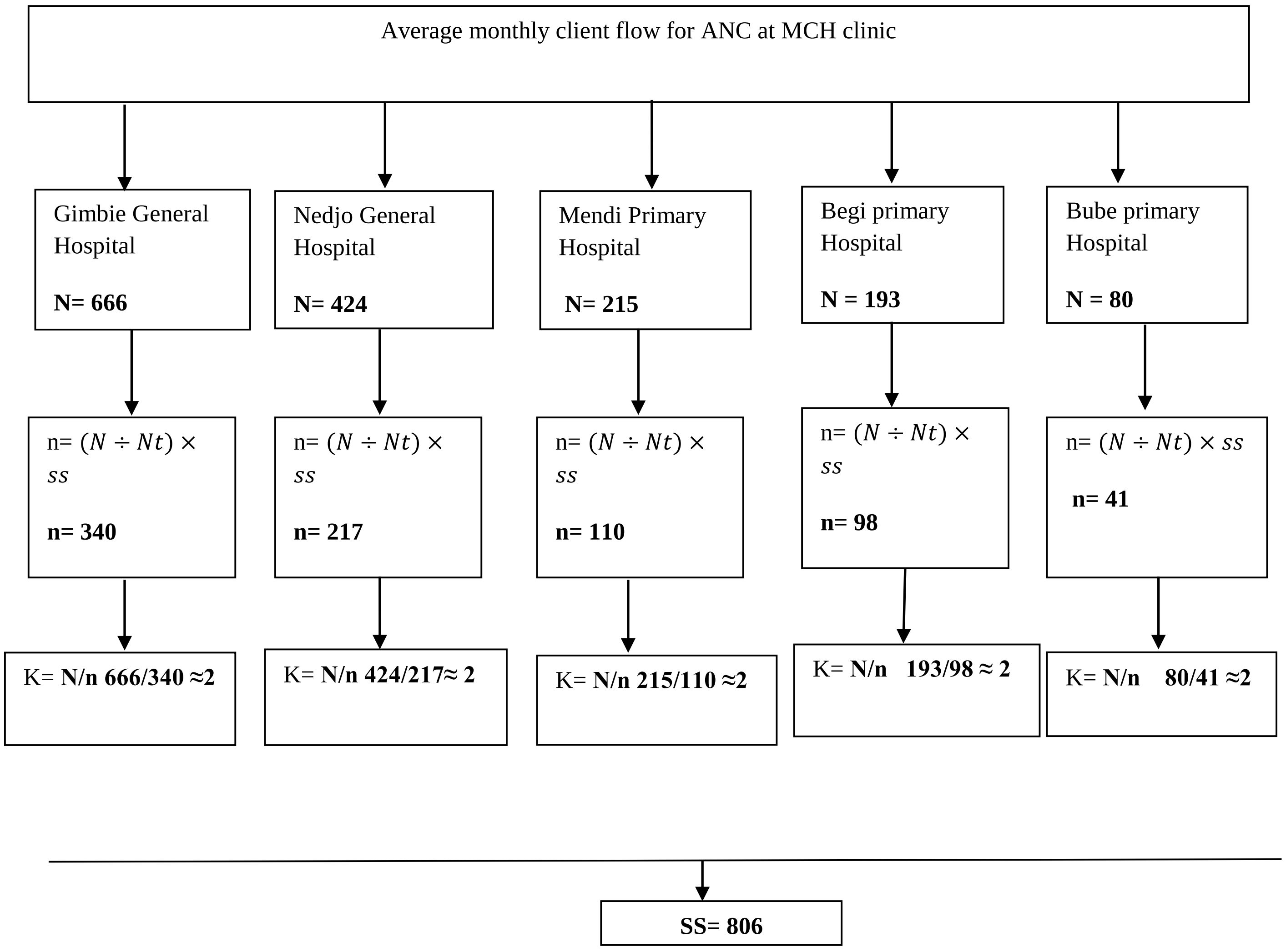
Average monthly client flow for ANC at MCH clinic
Gimbie General Hospital
N= 666
Nedjo General Hospital
N= 424
Mendi Primary Hospital
N= 215
Begi primary Hospital
N = 193
Bube primary Hospital
N = 80
n= (N ÷ Nt) × ss
n= 340
n= (N ÷ Nt) × ss
n= 217
n= (N ÷ Nt) × ss
n= 110
n= (N ÷ Nt) × ss
n= 98
n= (N ÷ Nt) × ss
n= 41
K= N/n 666/340 ≈2
K= N/n 424/217≈ 2
K= N/n 215/110 ≈2
K= N/n 193/98 ≈ 2
K= N/n 80/41 ≈2
SS= 806

Fig 1.

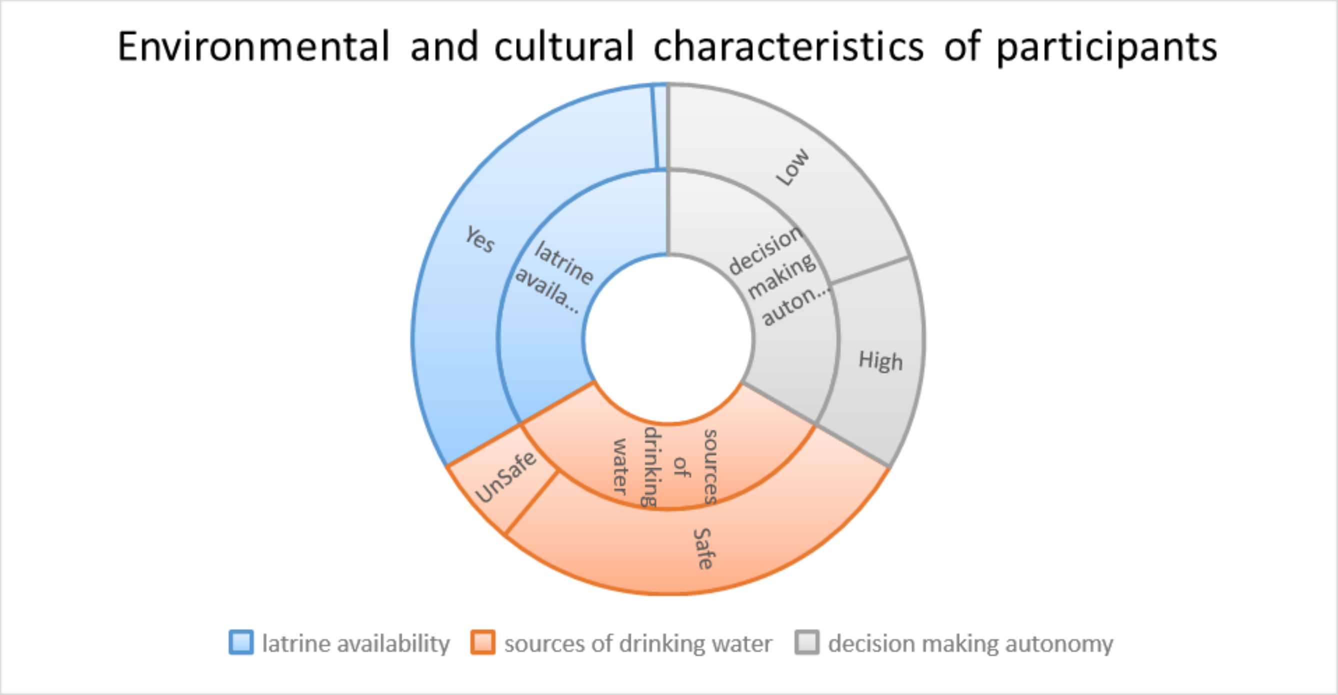
Environmental and cultural characteristics of participants
Low
decision
making
auton...
High
Yes
latrine
availa...
UnSafe
sources
of
drinking
water
Safe
latrine availability
sources of drinking water
decision making autonomy

Fig 2.

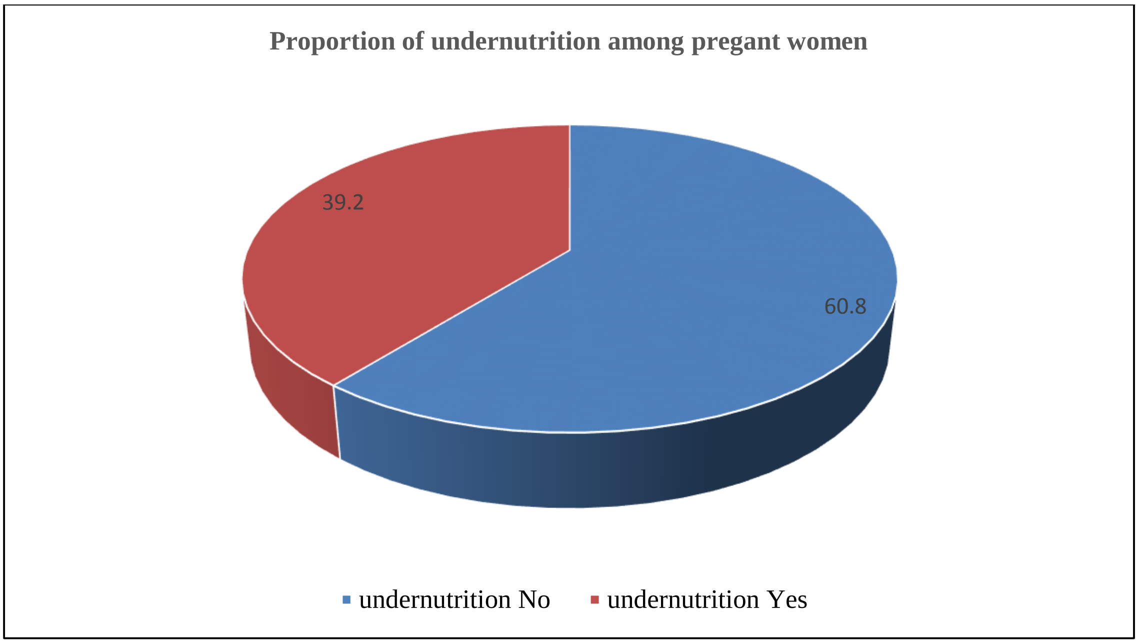
Proportion of undernutrition among pregant women
39.2
60.8
undernutrition No
undernutrition Yes

Fig 3.

Click here to access/download
Supporting Information
S1 Annex. Study Questionnaire in English.docx
