# Supplementary material for: The prevalence of under nutrition and associated factors among pregnant women attending antenatal care service in public hospitals of western Ethiopia
Source: PLoS One. 2023 Jan 17;18(1):e0278180. doi: 10.1371/journal.pone.0278180 (PMC9844891; doi:10.1371/journal.pone.0278180)
Supplement: Attachment — Submitted filename: renamed_89655.docx [file pone.0278180.s004.docx]

Review -

The theme is relevant, however follow some suggestions.

Abstract:

The objective is described twice in the abstract

Key words: replace “under nutrition”, as it is already in the title.

Background: I

suggest finishing with the objective of the study.

Methods and materials:

Describe the location hospitals of the Oromia region.

Contextualizing about antenatal care visits, is always performed in hospitals, low and medium risk pregnant women receive the same standard of care, ...

It was not clear whether the pregnant women included were the ones who would have the delivery or only those who attended the antenatal care visits.

Acronym: ANC,MUAC, BSc

In the exclusion criteria explain better what they consider seriously sick.

Were high-risk pregnant women, for example with diabetes and/or hypertension included?

How was the classification of under nutrition performed only by the MUAC?

Were no other parameters used, such as the Growth Charts and Gestational Age?

I suggest justifying and referencing the use of MUAC more intensely, since it is not an indicator used for pregnant women and is not robust for under nutrition .

What does substance use mean?

What are dietary habits evaluated and classified in what way? Which reference authors? To assess household food insecurity there are instruments widely used in the literature.

Consumption of common food groups. Which authors used to classify it as adequate or inadequate?

Result: Excluiria a figura 3

In the methodology they mention hemoglobin level, but I did not find this information in the results

Discussion

line 258-259: The prevalence of malnutrition described in other studies used the same parameters, i.e., MUAC for pregnant women?

Conclusion:

...“nearly forty percent of pregnant women were undernourished”... – would add by MUAC indicator
